# Foods of animal origin: a prescription for global health

**DOI:** 10.1093/af/vfz036

**Published:** 2019-09-28

**Authors:** Eric P Berg

**Affiliations:** Department of Animal Sciences, North Dakota State University, Fargo, ND

Most often we associate malnutrition with undernutrition. The World Health Organization (WHO, 2019) describes undernutrition as wasting (low body weight relative to height), stunting (low height relative to age), and underweight (low body weight for a given age). However, the WHO also classifies malnutrition as inadequate or excess vitamins/minerals, overweight, and obesity. Anemia (iron and B12 deficiency) and indispensable amino acid malnutrition are physically manifested as wasting, stunting, and underweight as well as overweight and obesity.

This issue of *Animal Frontiers* is titled “Foods of Animal Origin: A Prescription for Global Health.” This phraseology was selected because “global health” can mean different things to different people. For example, it can mean the physical health of an individual to positively affect their ability to thrive, or global health can mean the environmental health of the planet and its ability to sustain all life. The balance between providing for human health without compromising environmental health must be addressed. This issue includes articles that provide evidence for consuming nutrient-dense foods of animal origin to prevent/cure the most common global nutrition-related conditions and articles that address the social, cultural, environmental, and ethical considerations of consuming animal-sourced foods (Figure 1).

**Figure 1.**
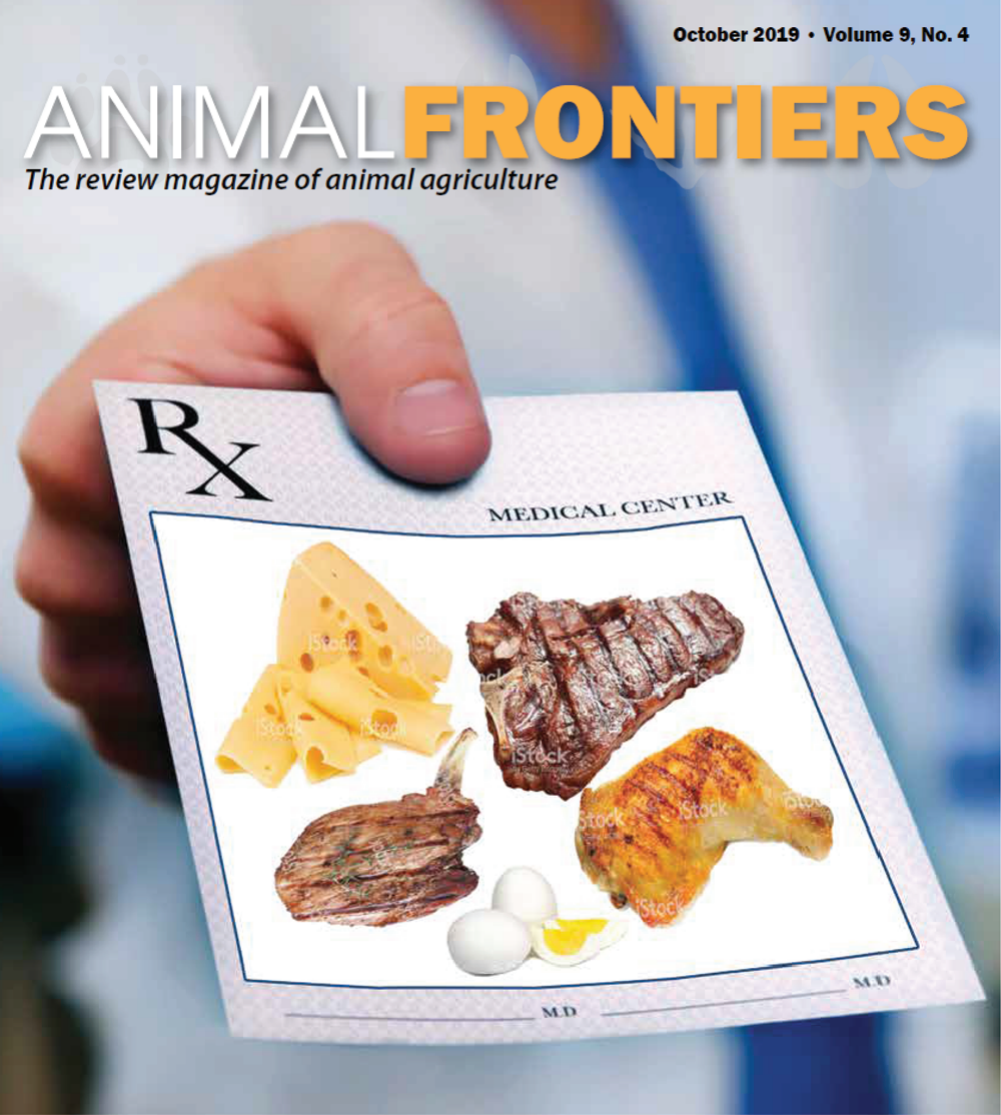
Schematic depicting examples of foods of animal origin as a prescription for global health.

The paper by Tang (2019) addresses the importance of correct food selection during the first 5 to 12 mo of age when solid foods are progressively introduced to infants. The foods selected for “complementary feeding” during this period can have a significant impact on food choices later in life as well as susceptibility to overweight/obesity and obesity-related metabolic disorders. The foods first introduced to children in transition are often cereal-based and low in high-quality protein with baby food meats being consumed the least. Anemia is a concern in early childhood and infant iron deficiencies may be prevented by the consumption of meat (especially “red” meat) as a complementary food. Furthermore, early research suggests that consumption of nutrient-dense muscle foods may provide beneficial substrates that promote development of a positive gut microbiome that becomes established during the transition period from breastmilk/formula to complementary foods.

Establishing good nutrition choices early in life provides the basis for lifelong dietary patterns that will improve an individual’s ability to thrive later in life. Hackney et al. (2019) describe the contribution of foods of animal origin for the prevention of muscle wasting later in life. The aging population often avoid meat (specifically “red” meat). As muscles age, there is a greater protein turnover and if protein degradation is greater than protein synthesis, then muscle tissue shrinks (metabolically termed wasting). Loss of muscle mass leads to loss of strength and greater susceptibility to falls and injury. Muscle serves as a metabolic sink for energy and a loss in muscle mass can also lead to development of obesity. Foods of animal origin are a high-quality source of highly digestible nutrients that can aid in maintenance of muscle health, providing more nutrients from fewer calories.

The Food and Agriculture Organization (FAO) convened a consortium of human nutrition experts that recommended (FAO, 2013): a) Dietary amino acids should be treated as individual nutrients whereby food labels should include information about digestible or bioavailability of individual amino acids and b) Quality of protein will be defined as a measure of digestible indispensable amino acid score (DIAAS). The FAO (2013) further stated that “Digestibility should be based on the true ileal digestibility of each amino acid preferably determined in humans, but if this is not possible, in growing pigs or in growing rats in that order (FAO, 2013).” Bailey and Stein (2019) describe the process for determination of true ileal digestibility of various animal-sourced foods. Most foods of animal origin are excellent sources of digestible protein, making them an important complimentary food with other foods possessing a lower DIASS, such as grains.

There is a growing ethical debate regarding how our food choices affect global environmental health. Alonso et al. (2019) present the case that meat, milk, and eggs consumed during the first 1,000 d of life (from conception, through pregnancy, to 2 yr of age) can significantly improve an individuals’ ability to thrive later in life. The authors also caution that as economic status improves and demand increases for foods of animal origin, history has shown that populations will demand greater quantities of that good thing. Left unregulated, this can lead to overproduction and abuse within the system that can include mistreatment of animals, land, natural resources, and people (employees).

On the surface, it may seem biologically impossible for obesity to be associated with malnutrition. Dasi et al. (2019) address the “double burden of malnutrition” where nutrient deficiencies lead to muscle wasting and a simultaneous increase in adiposity. In India, one in four urban adults over the age of 55 have been diagnosed with diabetes. Populations of low economic status often subsist on cereal-based diets deficient in such essential nutrients as vitamin B12, heme-iron, and many indispensable amino acids. Poor nutritional education in these parts of the world results in food choices that over-index starches (carbohydrates) and under-index high-quality proteins. Introduction of affordable animal sourced foods and nutrition education programs can improve the ability of these populations to thrive, especially for children.

We are often instructed to choose dietary patterns that include foods that are part of “sustainable” agriculture. Varijakshapanicker et al. (2019) address the balance between providing optimal nutrition and sustainability. The authors define sustainability as “a holistic concept that jointly considers ecological, social, and economic dimensions of a system or intervention for long-lasting prosperity.” They describe how livestock can be utilized in a sustainable system because they will consume plants that are not eaten by humans or they graze lands that are unable to sustain crops. Livestock are net contributors to human protein requirements because the grains, grasses, or forages they consume would not provide the same quality of bioavailable protein as animal-sourced foods. Furthermore, women play a very important role in livestock production in low- and middle-income countries. Management of such an economically important asset such as livestock has led to greater empowerment of women within the community.

A person’s ability to thrive in life is definitely dependent on their physical health, but mental health is also vital. Brain development, the ability to reason, make decisions, and improve intellectually all hinge on choosing the correct nutrients and combination of nutrients in a healthy diet. Balehegn et al. (2019) describe how the same dietary imbalances or deficiencies that lead to conditions of stunting and wasting may also impair cognitive development. Furthermore, animal-sourced foods provide more bioavailable sources of iron, zinc, iodine, and vitamins B12, B6, folate, and riboflavin that are necessary for proper brain development including enhancement of neural integrity and neural connectivity.

This issue of *Animal Frontiers* provides excellent insight into reaching a sustainable balance between providing optimal nutrition for a growing human population while maintaining the environmental harmony of the earth. Achieving this balance will require environmental stewards that are of strong body and mind. Foods of animal origin are nutrient-dense foods that should be the dietary staple throughout the lifecycle allowing humankind to not just survive, but to thrive.

## About the Author

**Eric Berg** is the Associate Department Head and Professor in the Department of Animal Sciences at North Dakota State University. His current research focus involves using swine as a biomedical model for humans to study the role of meat in a healthy diet. He is a Fellow of the American Meat Science Association and is currently serving on their Board of Directors as Past-President.
